# 2628. Prevalence of respiratory symptoms in children and staff in Pre-K—12^th^ grade schools and subsequent results from respiratory virus testing

**DOI:** 10.1093/ofid/ofad500.2241

**Published:** 2023-11-27

**Authors:** Jennifer Goldman, Jennifer E Schuster, Rangaraj Selvarangan, Olivia Almendares, Sadia Sleweon, Hannah L Kirking, Brian R Lee

**Affiliations:** Children's Mercy Hospital, Kansas City, Missouri; Children’s Mercy Kansas City, Kansas City, Missouri; Children’s Mercy Kansas City, Kansas City, Missouri; Centers for Disease Control and Prevention, Atlanta, Georgia; Center for Disease Control and Prevention, Atlanta, Georgia; Division of Viral Diseases, National Center for Immunization and Respiratory Diseases, CDC, Atlanta, Georgia; Children's Mercy Kansas City, Kansas City, Missouri

## Abstract

**Background:**

Little is known about the prevalence of respiratory symptoms and associated respiratory virus detection in students/ staff in primary and secondary schools.

**Methods:**

School KIDS is a prospective respiratory viral surveillance program in a large Missouri school district. Participating students/ staff complete a monthly electronic symptom survey that asks about respiratory symptoms (sx) in the preceding 7 days; participants are categorized based on the presence of any respiratory sx: ongoing (current), resolved, and no sx. Within 36 hours of survey completion, participants undergo surveillance respiratory viral testing at school. Self-administered nasal swabs are tested via multipathogen PCR assay (Table). Prevalence of respiratory viruses in participants with and without sx were calculated. A mixed-effect multivariable logistic regression model was used to compare odds of a sample testing positive based on surveillance sx and enrollee age.

**Results:**

A total of 544 students (30 pre-kindergarten (preK), 320 elementary, 117 middle, and 77 high) and 224 staff enrolled. A total of 2,537 sx surveys with corresponding respiratory samples were collected from November 2, 2022–April 15, 2023 (Table). Of the samples provided, 1,138 were preK-elementary (45%), 368 middle (14%), 276 high (11%), and 755 staff (30%). Participants reported ongoing (20%), resolved (14%), and no sx (66%). Prevalence of commonly reported sx were congestion (21%), runny nose (17%), cough (16%), and fever (3%). Overall respiratory virus test positivity was 26%; 50% of positive samples came from participants without sx. Flu A, PIV, and RSV were more frequently detected in those with ongoing sx. AdV, HMPV, RV/EV, and SARS-CoV-2 were more frequently detected in those with no sx. Increased odds of a positive test were observed among enrollees who reported runny nose (adjusted OR 1.4 [CI 1.0, 1.8]), congestion (adjusted OR 2.2 [CI1.6, 2.8]), cough (adjusted OR 1.5 [CI 1.1, 1.9]), or sore throat (adjusted OR 1.5 [CI 1.1, 2.0]) but not fever (adjusted OR 1.1 [CI 0.7,1.8]).

**Figure:**
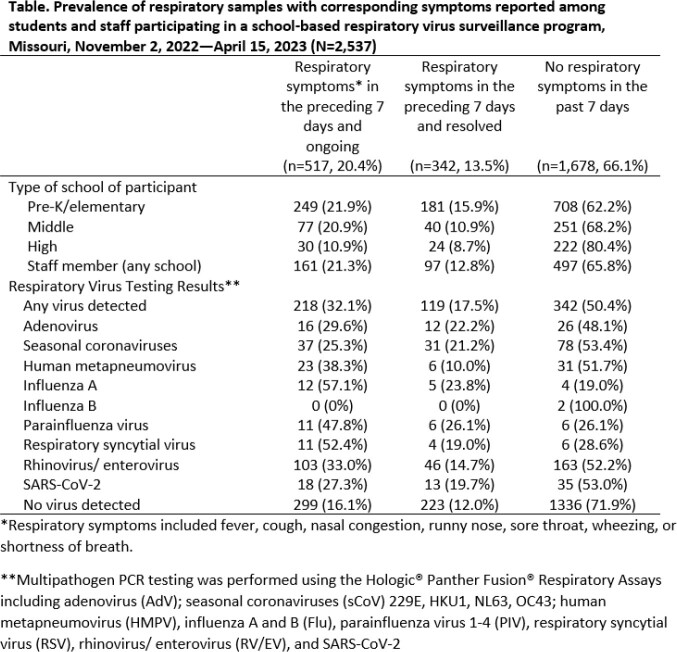

**Figure:**
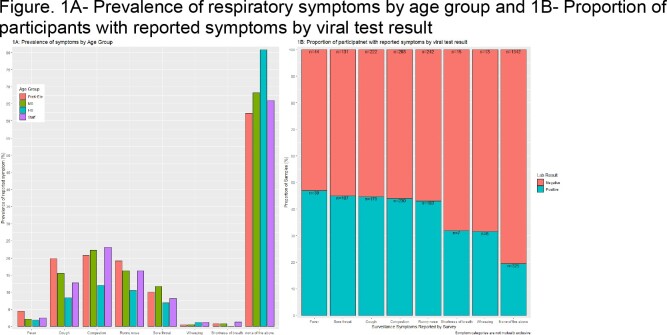

**Conclusion:**

More research is needed to understand if students with respiratory sx are more likely than asymptomatic students to shed transmissible respiratory viruses.

**Disclosures:**

**Rangaraj Selvarangan, BVSc, PhD, D(ABMM), FIDSA, FAAM**, Abbott: Honoraria|Altona Diagnostics: Grant/Research Support|Baebies Inc: Advisor/Consultant|BioMerieux: Advisor/Consultant|BioMerieux: Grant/Research Support|Bio-Rad: Grant/Research Support|Cepheid: Grant/Research Support|GSK: Advisor/Consultant|Hologic: Grant/Research Support|Lab Simply: Advisor/Consultant|Luminex: Grant/Research Support

